# A narrative review of current evidence supporting the implementation of electronic patient-reported outcome measures in the management of chronic diseases

**DOI:** 10.1177/20406223211015958

**Published:** 2021-05-24

**Authors:** Olalekan Lee Aiyegbusi, Devika Nair, John Devin Peipert, Kara Schick-Makaroff, Istvan Mucsi

**Affiliations:** Centre for Patient Reported Outcomes Research, Institute of Applied Health Research, University of Birmingham, Birmingham, B15 2TT, UK National Institute for Health Research (NIHR) Applied Research Centre, West Midlands, UK; Division of Nephrology and Hypertension, Vanderbilt University Medical Center, Nashville, TN, USA Vanderbilt O’Brien Center for Kidney Disease, Nashville, TN, USA; Department of Medical Social Sciences, Northwestern University Feinberg School of Medicine, Chicago, IL, USA; Faculty of Nursing, University of Alberta, Edmonton, Alberta, Canada; Multiorgan Transplant Program, University Health Network and Division of Nephrology, Department of Medicine, University of Toronto, ON, Canada

**Keywords:** Patient-reported outcomes, PROs, Quality of life, Outcome assessment, Electronic patient-reported outcomes, ePROs, ePROM systems, Digital health, Chronic diseases, Symptom monitoring

## Abstract

An application of telemedicine of growing interest and relevance is the use of personal computers and mobile devices to collect patient-reported outcomes (PROs). PROs are self-reports of patients’ health status without interpretation by anyone else. The tools developed to assess PROs are known as patient-reported outcomes measures (PROMs). The technological innovations that have led to an increased ownership of electronic devices have also facilitated the development of electronic PROMs (ePROMs). ePROMs are a conduit for telemedicine in the care of patients with chronic diseases. Various studies have demonstrated that the use of ePROMs in routine clinical practice is both acceptable and feasible with patients increasingly expressing a preference for an electronic mode of administration. There is increasing evidence that the use of electronic patient-reported outcome (ePROMs) could have significant impacts on outcomes valued by patients, healthcare providers and researchers. Whilst the development and implementation of these systems may be initially costly and resource-intensive, patient preferences and existing evidence to support their implementation suggests the need for continued research prioritisation in this area. This narrative review summarises and discusses evidence of the impact of ePROMs on clinical parameters and outcomes relevant to chronic diseases. We also explore recently published literature regarding issues that may influence the robust implementation of ePROMs for routine clinical practice.

## Introduction

Telemedicine refers to the use of electronic information and communications technology to provide and support healthcare remotely.^1,2^ An application of telemedicine of growing interest and relevance is the use of personal computers and mobile devices to collect patient-reported outcomes (PROs).^3^ PROs are self-reports of patients’ health status without interpretation by anyone else.^4^ The tools developed to assess PROs are known as patient-reported outcomes measures (PROMs).

Although the use of PROMs in clinical trials to assess the impact of interventions is well established, interest in their use in routine clinical practice is on the increase. The technological innovations that led to an increased ownership of electronic devices have also facilitated the development of electronic PROMs (ePROMs).^5^ PROMs are traditionally paper-based while the term ‘ePROMs’ refers to telephone-based interactive voice response systems and screen-based systems.^6^ ePROMs may be administered at clinic, with or without clinical supervision, or remotely in an unsupervised setting (such as subject’s home or workplace). Remote assessment enables more timely and accurate self-reporting and may avoid potential recall bias.^6^

The National Centre for Chronic Disease Prevention and Health Promotion (NCCDPHP) defined chronic diseases as conditions that last a year or more and require ongoing medical attention or limit activities of daily living or both.^7^ The definition encompasses conditions such as heart diseases, cancer, chronic lung disease, stroke, Alzheimer’s disease, diabetes and chronic kidney disease (CKD). Most of these diseases are presently incurable. Patients with chronic diseases often require long-term care while experiencing significant symptom burden, which may impair their health-related quality of life (HR-QOL).^8^

The use of ePROMs provides patients the opportunity to report remotely the impact of disease and treatment on their HR-QOL. Such information may inform the clinical management of patients. Various studies have demonstrated that the use of ePROMs in routine clinical practice is both acceptable and feasible, with patients increasingly expressing a preference for an electronic mode of administration.^9,10^

However, despite these advancements, doubts about the quantifiable benefits of implementing PROMs/ePROMs for use in routine clinical practice persist and there are still concerns around integration with existing workflows.^11–13^ Whilst the potential impact of PROMs/ePROMs on patient-clinician communication has been well documented,^14–16^ their impact on patient outcomes or clinical parameters has been limited.^17,18^

A systematic review by Boyce and Browne found weak evidence that provision of PROM data to healthcare professionals resulted in a positive impact on patient outcomes.^19^ The review also reported that studies that demonstrated the most significant impact used PROMs to facilitate patient care in an outpatient setting and there was weak evidence supporting their use as a screening tool.^19^ The review included commentaries, editorials, systematic reviews and a limited number of primary studies.

The majority of the relevant primary studies available at the time of Boyce and Browne’s review reported PROM projects in their nascent stages.^20–24^ These studies were mostly cross-sectional, feasibility or pilot studies, which are not designed to capture the impact of PROM data on patient outcomes. Furthermore, these studies were conducted prior to the publication of PRO-specific clinical trial guidelines such as the SPIRIT-PRO (Standard Protocol Items: Recommendations for Interventional Trials-PRO Extension) to facilitate the incorporation of PROs into clinical trial protocols and the CONSORT-PRO (Consolidated Standards of Reporting Trials-PRO Extension) to guide the reporting of PROM data from clinical trials.^25,26^

Since the publication of the Boyce and Browne review in 2013, an increasing number of high quality, longitudinal studies aimed at measuring the impact of ePROMs on clinical outcomes have been reported.^27,28^

This review aimed to summarise and discuss evidence of the impact of ePROMs on clinical outcomes relevant to the management of chronic diseases as defined by the NCCDPHP. We also explored recently published literature regarding issues that may influence the robust implementation of ePROMs for routine clinical practice. We focus on articles published since the Boyce and Browne 2013 review.

## Methods

### Search strategy

Prior to conducting this review, the authors were aware of the two highly cited articles by Basch *et al.* that reported compelling evidence of the impact of ePROMs on clinical outcomes since the publication of the Boyce and Brown review.^19,28,29^ Forward citation searches of these three articles on the Web of Science (WoS) database (All Databases version) were conducted by OLA on 12 September 2020.^19,28,29^ This approach facilitated the efficient retrieval of recent and relevant articles. In addition, Google Scholar was searched on 12 February 2021 to ensure that all relevant and recent articles were captured.

### Screening process

Titles and abstracts were screened for relevance and full-text articles were obtained for articles that potentially met the eligibility criteria.

### Eligibility criteria

Randomised controlled trials (RCTs), cohort studies and observational studies of any chronic disease were included if they reported the impact of ePROMs on clinical outcomes and/or healthcare resource use in chronic disease care. Specifically, our outcomes of interest included: patient survival, symptom management, treatment adherence and utilisation of healthcare resources. There were no language restrictions.

Articles were excluded if they reported: (i) studies relating to PROMs administered in paper form; (ii) clinician-reported instruments; (iii) pilot studies that only detailed the feasibility or acceptability of ePROMs or impacts on patient-clinician communications; and (iv); editorials, reviews, and conference abstracts.

### Critical appraisal

The included studies were appraised using the appropriate critical appraisal skills programme (CASP) checklists.^30^

## Findings

The search on WoS retrieved 1099 entries for title and abstract screening. The full-texts for 52 were obtained for further review and 16 articles selected. Table 1 summarises the characteristics and findings of the included articles. Most of the articles reporting the impact of ePROMs on clinical parameters and outcomes in chronic conditions were derived from oncology. A few focussed on diseases such as rheumatoid arthritis, epilepsy and sleep apnoea.^27,31^ All except one study were conducted in outpatient settings and most were RCTs.^32^ There was evidence that the use of ePROMs in routine clinical practice may improve patient survival, symptom management and individualised care, treatment adherence; encourage efficient utilisation of healthcare resources; and reduce risk of disease transmission during outbreaks and epidemics. A critical appraisal of the studies was conducted and the findings summarised in Table 2.

**Table 1.** Studies reporting the impacts of ePROs on patient outcomes, utilisation of resources and in chronic disease care.

Study	Setting, country	Design	Patient group	ePROM intervention, sample size (*n*)	Usual care (control), sample size (*n*)	Outcome(s) of interest	Clinical interventions to ePROM data	Result(s)
Absolom *et al*.^33^	Outpatient, tertiary care providing chemotherapy	Prospective, randomised two-arm parallel group study	Patients initiating systemic treatment (chemotherapy with or without targeted therapies) for colorectal, breast, or gynaecological cancers.	eRAPID was added to usual care. Participants completed online symptom questions from home over 18 weeks (at least weekly plus when having symptoms). Reminders were sent weekly *via* text or e-mail. (*n* = 256)	Patients were regularly assessed by oncologists or nurses in clinics or by telephone for toxicity and to prescribe next treatment. Patients contacted the hospital *via* a 24/7 emergency hotline. (*n* = 252)	(a) Symptom control at 6, 12, and 18 weeks after baseline.(b) Impacts on hospital services (process of care measures)	Participants received immediate advice on symptom management or a prompt to contact the hospital. The symptom reports were displayed in real time in EPR. Nurses monitored email alerts for severe symptoms.	• Based on FACT-PWB scores, positive effects of eRAPID were observed at 6 (*p* = 0.028) and 12 weeks (*p* = 0.039), but there was no significant difference at the primary end point of 18 weeks (*p* = 0.69) • No between-arm differences were found for chemotherapy delivery, hospital admissions, acute oncology assessments, or emergency hotline calls
Basch *et al*.^28^	Outpatient providing tertiary care and chemotherapy, US	Single-centre, non-blinded RCT	Advanced solid tumours - metastatic breast, genitourinary, gynaecologic, or lung cancers	STAR system for symptom monitoring. Includes patient adapted questions from NCI-CTCAE. Email reminders were sent. Computer-experienced (*n* = 286), computer-inexperienced (*n* = 155)	Symptoms discussed during clinic. Patients encouraged to telephone between visits for concerning symptoms. Computer-experienced (*n* = 253), computer-inexperienced (*n* = 72)	(i) Survival(ii) Quality-adjusted survival(iii) ED visits(iv) HospitalisationAt 1 year for the entire study group and subgroups.	Nursing response to alerts: (i) Telephone for symptom management counselling(ii) Medication initiation/change(iii) Referrals(iv) Chemotherapy dose modification(v) Imaging/test orders	• Survival: STAR *versus* usual care (75% *versus* 69%; *p* = 0.05) • Survival: computer-inexperienced STAR *versus* usual care (74% *versus* 60%; *p* = 0.05) • ED visits: STAR *versus* usual care (34% *versus* 41%; *p* = 0.02) • Quality-adjusted survival, STAR *versus* usual care (mean: 8.7 months *versus* 8.0 months; *p* = 0.004) • ED visits: computer inexperienced subgroups, STAR *versus* usual care (34% *versus* 56%; *p* = 0.02) • Hospitalisation: STAR *versus* usual care (45% *versus* 49%; *p* = 0.08) • Hospitalisation: computer-inexperienced subgroup, STAR *versus* usual care (44% *versus* 63%; *p* = 0.003)
Basch *et al*.^29^	Outpatient, US	Single centre, non-blinded RCT; follow-up analysis	Advanced solid tumours - metastatic breast, genitourinary, gynaecologic, or lung cancers	STAR system.	Symptoms discussed during clinic. Patients encouraged to telephone between visits for concerning symptoms.	OS at median follow up of 7 years	Nursing response to alerts: (i) Telephone for symptom management counselling(ii) Medication initiation/change(iii) Referrals(iv) Chemotherapy dose modification(v) Imaging/test orders	Median OS: STAR (31.2 months; 95% CI, 24.5–39.6) *versus* usual care (26.0 months; 95% CI, 22.1–30.9) (difference, 5 months; *p* = 0.03)
Denis *et al*.^34^	Outpatient, France	Single centre, phase II trial	Patients with surgical excision, complete response, or detectable but non-progressive lung carcinoma	Sentinel PRO system. Weekly reports on 11 symptoms. (*n* = 49)	Clinic visit and imaging every 2–6 months according to tumour stage and treatment type.	Survival	Phone call to confirm symptoms. Follow-up visits and imaging subsequently organised.	• Median survival: Sentinel (22.4 months) *versus* usual care (16.7 months), (*p* = 0.0014). • One-year survival: Sentinel (86.6%) *versus* usual care (59.1%)
Denis *et al*.^35^	Outpatient, France	Multicentre phase III randomised trial	Advanced stage IIA (TXN1) to IV, non-progressive small cell or non–small cell lung cancer. Post treatment or on maintenance chemotherapy.	Sentinel PRO system. Weekly reports on 12 symptoms. (*n* = 60)	Routine follow up with CT scans scheduled every 3–6 months according to disease stage. Patients were encouraged to call between visits if they had new or progressive symptoms. (*n* = 61)	(i) OS(ii) PFSMedian follow up 9 months.	Alerts prompted by the Sentinel PRO system were confirmed by a phone call from the oncologist and led to an unscheduled visit.	• Median OS: Sentinel, 19.0 months (95% confidence interval [CI] = 12.5 to non-calculable) *versus* usual care, • 12.0 months (95% CI = 8.6–16.4), (one-sided *p* = 0.001). • HR = 0.32 [95% CI = 0.15–0.67]; one-sided *p* = 0.002). • PFS was not statistically significantly different between the two arms (*p* = 0.13). • Rate of imaging reduced 49% per patient per year in Sentinel *versus* usual care.
Denis *et al*.^36^	Outpatient, France	Multicentre phase III randomised trial.Final overall analysis; 10 patients who received usual care had not relapsed and crossed over to ePRO intervention.	Advanced stage IIA (TXN1) to IV, non-progressive small cell or non–small cell lung cancer. Post treatment or on maintenance chemotherapy.	Sentinel PRO system. Weekly reports on 13 symptoms.	Routine follow-up with CT scans scheduled every 3–6 months according to disease stage. Patients were encouraged to call between visits if they had new or progressive symptoms.	OS after 2 years of follow up	Alerts prompted by the Sentinel PRO system were confirmed by a phone call from the oncologist and led to an unscheduled visit.	• Median OS without censoring for crossover: Sentinel (22.5 months) *versus* usual care (14.9 months) HR = 0.59 [95%CI, 0.37–0.96]; *p* = 0.03) • Median OS with censoring for crossover: Sentinel (22.5 months) *versus* usual care (13.5 months) HR = 0.50 [95% CI, 0.31–0.81]; *p* = 0.005) • Death: 29 (47.5%) in Sentinel and 40 (66.7%) in usual care
de Thurah *et al*.^31^	Outpatient, Denmark	Pragmatic noninferiority RCT at two centres.	Rheumatoid arthritis	AmbuFlex ePRO system used as a decision aid in deciding whether patients required an outpatient appointment. The 11-item Flare-RA was used.Arm 1: Follow up by a rheumatologist (PRO-TR) (*n* = 93)Arm 2: Follow up by a nurse (PRO-TN) (*n* = 88)	Physicians saw patients in the outpatient clinic every 3–4 months. (*n* = 94)	Change in DAS28 after week 52.	Patients in the AmbuFlex groups were seen in outpatient clinic if their Flare-RA score was ⩾2.5 and/or their C-reactive protein (CRP) level was ⩾10 mg/litre.	• Noninferiority was established for the DAS28 in both AmbuFlex groups when compared with usual care. In the ITT analysis: • Mean differences in the DAS28 score between PRO-TR *versus* usual care = −0.10 ([90% CI]:−0.30, 0.13) • Between PRO-TN *versus* usual care = -0.19 (90% CI: -0.41, 0.02). • Including 1 yearly visit to the outpatient clinic patients in: • PRO-TN: 1.72 ± 1.03 visits/year • PRO-TR: 1.75 ± 1.03 visits/year • Usual care: 4.15 ± 1.0 visits/year.
Fjell *et al*.^37^	Outpatient, Sweden	non-blinded RCT at two centres	Patients with breast cancer undergoing neoadjuvant chemotherapy.	The Interactive ‘Interaktor’ ePRO application for symptom reporting, self-care advice and symptom management during neoadjuvant chemotherapy. Utilised 14 questions. Email reminders were sent. (*n* = 74)	(i) Outpatient visits before each chemotherapy treatment(ii) A visit where the treatment, related symptoms and how to manage them were discussed.(iii) Contact number for a nurse for treatment related concerns (*n* = 75)	Symptom burden two weeks after completing chemotherapy	Patients had continuous access to evidence-based self-care advice and relevant websites. An alert triggered a notification suggesting to the patient related self-care advice. An alert also initiates a phone call from a nurse.	Using the MSAS questionnaire, Interaktor group had: • Lower prevalence of nausea (*p* = 0.041), vomiting (*p* = 0.037), and feelings of sadness (*p* = 0.003) • Less overall symptom distress (*p* = 0.004), and physical symptom distress (*p* = 0.031). • Lower scores in the total MSAS (*p* = 0.033). Effect size = 0.26– 0.34.Using the EORTC QLQ-C30, Interaktor group had: Higher emotional functioning (*p* = 0.008)
Handa *et al*.^38^	Outpatient, Japan	Single centre RCT	Patients undergoing breast cancer chemotherapy.	The BPSS smartphone/PC application. A support tool for supportive management for adverse drug reactions.Patients’ symptoms were recorded using the CTCAE v4.0. (*n* = 47)	(i) Patients received explanatory materials compiled by drug manufacturer(ii) Medical staff recommended patients record their progress using their own notes. (*n* = 48)	Change in HADS score baseline and after completion of 4 chemotherapy courses	None reported	For the BPSS app group there were no significant differences in HADS: Anxiety: 1.66 (95% CI, 0.92–2.40)Depression: 0.09 (95% CI, −0.70–0.87).
Kricke *et al*.^39^	Outpatient, US	Single centre, observational study	Patients with suspected or confirmed SARS-CoV-2 infection	A daily electronic symptom and coping questionnaire. *n* = 6006 completed at least a questionnaire.	Not applicable	(i) Track illness(ii) Offer support(iii) Identify worsening patients	The programme used text message reminders, and telephone-based care. Members of the medical team evaluated symptom severity, provided information, and referred patients with severe symptoms to the ED.	• Of those who filled out a questionnaire, on any given day, about 20% reported concerning symptoms. • An average of 9 patients per day went to the ED (SD: 5; range: 1–21)
Kroenke *et al*.^40^	Outpatient, US	Multicentre, RCT	Patients screened positive for at least one SPADE (sleep, pain, anxiety, depression, and low energy/fatigue) symptom (their underlying chronic conditions were unspecified)	Participants electronically completed the PROMIS profile-29. Feedback of their symptom scores were provided to clinicians. (*n* = 150)	Participants also electronically completed the PROMIS profile-29. No feedback was provided to clinicians (*n* = 150)	(i) 3-month change in composite SPADE score	Clinicians received symptom scores for patients in the feedback group	• Non-significant trend favouring the feedback compared with control group (between-group difference in composite T-score improvement, 1.1; *p* = 0.17).
Mooney *et al*.^41^	Outpatient, US	Multicentre, RCT	Patients starting chemotherapy (underlying diagnosis unspecified).	Patients in the Symptom Care at Home (SCH) arm called the automate system daily to report symptom severity. They received self-management coaching. Alerts of poorly controlled symptoms were sent to nurses who intervened following a decision support guidance. (*n* = 180)	Patients also called the automate system daily to report symptom severity daily but did not receive any further interventions (*n* = 178)	• Symptom severity across all symptoms • Number of severe, moderate symptom days • Individual symptom severity	Patients in the SCH arm received self-management coaching. Alerts of poorly controlled symptoms were sent to nurses who intervened following a decision support guidance.	• Symptom severity across all symptoms was significantly less among SCH participants (*p* < 0.001). • Average reduction of symptom severity for SCH arm was 3.59 points (*p* < 0.001), roughly 43% of usual care. • SCH arm had significant reductions in severe (67% less) and moderate (39% less) symptom days compared with usual care (both *p* < 0.001). • All individual symptoms, (except diarrhoea), were significantly lower in the SCH arm (*p* < 0.05).
Nipp *et al*.^32^	Inpatient setting, US	Single centre, non-blinded, pilot RCT	Patients with advanced cancers who have unplanned hospital admissions	The IMPROVED monitoring system was used by participants to report daily symptoms. (*n* = 75)	Participants also reported their symptoms each day using tablet computers. Clinicians did not receive their symptom reports. (*n* = 75)	Preliminary efficacy of IMPROVED for: (i) Improving symptom burden(ii) Health care utilisation among hospitalised patients with advanced cancer.	The oncology team received the symptom reports for patients on IMPROVED and discussed these during morning rounds. Responses to the ePRO data were based on clinical judgement.	• Patients assigned to IMPROVED had a greater proportion of days with lower psychological distress: 0.12 (95% CI, 0.03– 0.21; *p* = 0.008). • Patients assigned to IMPROVED experienced improvements in their average symptom scores for drowsiness (B = −0.54, 95% CI: −1.04 to −0.05; *p* = 0.033) and shortness of breath (B = −0.43, 95% CI: −0.75 to −0.11; *p* = 0.009), but not for other individual physical symptoms. • No significant intervention effects on patients’ hospital length of stay.
Nipp *et al*.^42^	Outpatient, US	Secondary analysis of data from the STAR RCT by Basch *et al*.	Advanced solid tumours - metastatic breast, genitourinary, gynaecologic, or lung cancers	STAR system.	Symptoms discussed during clinic. Patients encouraged to telephone between visits for concerning symptoms.	To explore the moderating effects of age on the outcomes of the STAR RCT namely:	Nursing response to alerts: (i) telephone for symptom management counselling(ii) medication initiation/change (iii) referrals (iv) chemotherapy dose modification (v) imaging/test orders(i) Survival(ii) ER visits(iii) Hospitalisation	• Younger patients (median age = 58 years) on STAR experienced lower risk of ER visits (HR = 0.74, P = 0.011) and improved survival (HR = 0.76, P = 0.011) compared with younger patients on usual care. • Older patients (median age = 75 years) did not experience significantly lower risk of ER visits (HR = 0.90, P = 0.613) or improved survival (HR = 1.06, P = 0.753) with the intervention. • There were no moderation effects based on age for HRQOL • and risk of hospitalisation.
Rasschaert *et al.*^43^	Outpatient, Belgium	Multi-centre, prospective cohort	Patients with solid tumours (Gastro-intestinal, genito-urinary, breast, ling, head and neck, melanoma, gynae) receiving systemic antineoplastic agent(s) at any stage of their disease.	AMTRA was used for daily symptom reporting. Questions were drawn from PRO-CTCAE. A compliance tool to monitor oral therapies was incorporated in the system. Reminders were sent. (*n* = 168)	There was no control arm. Usual care included consultation with an oncologist during systemic treatment.Patients were provided information on treatment, toxicities and instructions on contacting the hospital for serious AEs.	Effect on:(i) symptom burden(ii) medication compliance	Staff received detailed training on the system and pathways generated by alerts. For toxicities graded as 1 or 2, self-management information was provided. For severe AE’s, medical staff received emailed alerts, contacted patients to advise or refer to the hospital.	• A reduction of mean grade over time for five toxicities (nausea, constipation, loss of appetite, fatigue, and dyspnea), (*p* < 0.0001). • Compliance to oral chemotherapy was high using AMTRA, median = 98.7% (95% CI: 93.5–100.0%).
Schougaard *et al*.^27^	Outpatient, Denmark	Multi-centre, observational implementation study	Patients with epilepsy, coronary heart disease, narcolepsy, sleep apnoea, prostate cancer, asthma, rheumatoid arthritis, colorectal cancer, and renal failure	AmbuFlex system, where the patients’ ePROs determined the need of an outpatient consultation. Items were *ad hoc*.Reminders were sent.	Not relevant as system has been implemented for routine use	Impact on utilisation of resources in epilepsy and sleep apnoea clinics	A traffic light alert system was developed.A clinician has to decide whether further contact is needed.Red: Definite need of contact or the patient asks for a consultation.	• Of 8256 telePRO-based contacts from epilepsy outpatients, up to 48% were handled without further contact. • For sleep apnoea up to 57% of the 1424 telePRO-based contacts did not require further contact. • Completion rates were over 90% at baseline and follow up.

AE, adverse event; AMTRA, ambulatory monitoring of cancer therapy using an interactive application; BPSS, breast cancer patient support system; CI, confidence interval; CT, computerized tomography; CTCAE, common terminology criteria for adverse events; DAS28, disease activity score in 28 joints; ED, emergency department; EORTC QLQ-C30, European organisation for research and treatment of cancer quality of life questionnaire C30; EPR, electronic patient records; ePRO, electronic patient-reported outcome; ER, emergency room; FACT-PWB, functional assessment of cancer therapy scale-general physical well-being subscale; HADS, hospital anxiety and depression scale; HR, hazard ratio; HRQOL, health-related quality of life; IMPROVED, improving management of patient-reported outcomes *via* electronic data; MSAS, memorial symptom assessment scale; NCI-CTCAE, National Cancer Institute’s common terminology criteria for adverse events; PFS, progression free survival (defined as the duration from randomisation to the first radiologic observation of disease progression or to last follow up when the patient is censored); PRO, patient-reported outcome; PROMIS, patient reported outcome measure information system; RA, rheumatoid arthritis; RCT, randomised controlled trial; SARS-CoV-2, severe acute respiratory syndrome coronavirus 2; STAR, symptom tracking and reporting; US, United States.

**Table 2.** Critical appraisal of included studies.

Articles	Study type	1	2	3	4	5	6	7	8	9	Comments
Absolom *et al*.^33^	RCT	✔	✔	✔	±	✔	✔	✔	✔	±	• Impossible to randomise clinicians who saw patients in both arms
Basch *et al*.^28^	RCT	✔	✔	✔	±	✔	✔	✔	✔	±	• Weekly e-mail reminders sent
											• Clinicians responded to alerts but had no specific guidance
Basch *et al*.^29^	Overall analysis	✔	✔	✔	±	✔	✔	✔	✔	±	• Post hoc analysis of data collected during the RCT^28^
Denis *et al*.^34^	Retrospective study	✔	🛇	✔	±	✔	✔	✔	✔	±	• Retrospective study in which the experimental and the control arms were not recruited at the same time.
											• Follow up durations were different for the two arms but not significant (*p* = 0.27)
											• Dependent on the quality of record keeping
Denis *et al*.^35^	RCT	✔	✔	✔	±	✔	✔	✔	✔	±	• Baseline characteristics were similar, but mean baseline FACT-L score higher in the experimental than in the control arm (*p* = 0.01)
											• After a pre-planned interim analysis in which a significant survival improvement was observed, there was mandatory cessation of recruitment and crossover of control patients to the intervention.
											• Inclusion of patients on maintenance therapy, who might have had increased interactions with care teams.
Denis *et al*.^36^	Overall analysis	✔	✔	✔	✔	✔	✔	✔	✔	±	• Post hoc analysis of data collected during the RCT^35^
de Thurah *et al*.^31^	RCT	✔	✔	✔	±	✔	✔	✔	✔	±	• Follow up assessment was performed randomisation by a blinded independent assessor.
Fjell *et al*.^37^	RCT	✔	✔	✔	±	✔	✔	✔	✘	±	• CIs were not reported making it difficult to ascertain the precision of the intervention effect.
Handa *et al*.^38^	RCT	✔	✔	✔	±	✔	-	✔	✔	±	• It was unclear what constituted usual care and whether the materials provided to the non-ePROM group was also given to ePROM group.
Kricke *et al*.^39^	Observational study	✔	🛇	✔	🛇	🛇	🛇	🛇	🛇	±	• This was an observational study.
											• No suitable checklist found to appraise this type of study therefore only questions 1, 3 and 9 from the CASP checklist for RCTs were used.^a^
											• Impossible to know whether there were statistically significant impacts on outcomes
											• Higher risk of bias
Kroenke *et al*.^40^	RCT	✔	✔	✔	±	✔	✔	✔	✘	±	• CIs were not reported making it difficult to ascertain the precision of the intervention effect.
											• Effect sizes were calculated for the intervention
Mooney *et al*.^41^	RCT	✔	✔	✔	±	✔	✔	✔	✘	±	• CIs were not reported making it difficult to ascertain the precision of the intervention effect.
											• Effect sizes were calculated for the intervention
Nipp *et al*.^32^	RCT	✔	✔	✔	±	±	✔	✔	✔	±	• It was not stated whether the differences in baseline data were significant or not.
											• Clinicians did not receive specific guidance about symptom management
Nipp *et al*.^42^	Secondary analysis	✔	✔	✔	±	±	✔	✔	✘	±	• Secondary analysis of RCT data^42^
											• Comparison of baseline characteristics was done by age group and not by study arm making it impossible to compare the study arms directly
											• CIs were not reported making it difficult to ascertain the precision of the intervention effect.
Rasschaert *et al*^43^	Cohort analysis	✔	🛇	✔	🛇	🛇	🛇	✔	✔	±	• Prospective cohort analysis
											• No suitable checklist found to appraise this type of study therefore only questions 1, 3, 7, 8 and 9 from the CASP checklist for RCTs were used.
Schougaard *et al*.^27^	Observational study	✔	🛇	🛇	🛇	🛇	🛇;	🛇;	🛇	±	• Observational study
											• No suitable checklist found to appraise this type of study therefore only questions 1, 7, 8 and 9 from the CASP checklist for RCTs were used.
											• Higher risk of bias

✔, Yes

✘, No

🛇, Not applicable.

±, Cannot tell

a CASP RCT checklist questions

1. Did the study address a clearly focussed research question?

2. Was the assignment of participants to interventions randomised?

3. Were all participants who entered the study accounted for at its conclusion?

4. Were the people analysing outcomes ‘blinded’?* (due to the nature of the intervention, patient or investigator blinding was impossible)

5. Were the study groups similar at the start of the randomised controlled trial?

6. Apart from the experimental intervention, did each study group receive the same level of care (that is, were they treated equally)?

7. Were the effects of intervention reported comprehensively?

8. Was the precision of the estimate of the intervention or treatment effect reported?

9. Do the benefits of the experimental intervention outweigh the harms and costs?

CASP, critical appraisal skills programme; CI, confidence interval; ePROM, electronic patient-reported outcomes measures; FACT-L, *Functional Assessment of Cancer Therapy*-Lung; RCT, randomised controlled trial.

## Evidence to support the use of ePROMs in routine clinical care

### Improved patient survival

A single-centre RCT by Basch *et al.* in the United States (US) reported statistically significant associations between the use of the ‘STAR’ (Symptom Tracking and Reporting) system, a web-based ePROM interface for tele-monitoring, and patient survival following chemotherapy for advanced solid tumours.^28^ The system incorporates ‘patient-adapted questions’ from the National Cancer Institute’s Common Terminology Criteria for Adverse Events (CTCAE) checklist.^44^ It was not clear from the article whether these questions were derived from CTCAE items or the patient-specific PRO-CTCAE.^45^

At 1 year after the start of the intervention, patients in the intervention arm, whose symptoms were monitored using STAR, experienced a significant survival advantage over those in the control arm who received routine surveillance.^28^ Email reminders were sent to patients randomised to the STAR group and nurses initiated clinical response to alerts generated by patients using the STAR system (see Table 1). The effect of the intervention on survival was greater among participants who had less computer experience, and who were more likely to be older, male, black and less educated.^28^ In an assessment of overall survival, conducted after a median follow-up of 7 years, patients in the STAR arm experienced a statistically significant survival advantage of 5 months over patients in the usual care arm.^29^

In a multicentre study conducted in France by Denis *et al.* that further tested the approaches employed by Basch *et al.*, patients with advanced lung cancer following initial treatment were randomly assigned to receive either web-based symptom monitoring *via* an ePROM system (Hyperion) or standard follow up (scheduled imaging).^35^ Interim analysis at 9 months showed that patients in the ePROM group experienced a survival benefit of 7 months. This led to the decision by the independent data monitoring committee to mandate the crossover of patients in the control to the intervention arm. Furthermore, the overall survival rate at 1 year was significantly higher in the ePROM arm than in the control arm (74.9% *versus* 48.5%).^35^ These results were attributed to earlier detection of lung cancer relapse in the ePROM group. In addition, as the ePROM system was reliable in detecting patient relapse, a 49% reduction in CT scans was reported for patients in the ePROM group. Analysis of median overall survival after 2 years of follow up reported that significant survival benefit persisted [22.5 months (ePROM group) *versus* 13.5 months (control group)].^36^

However, these results must be interpreted with caution. Survival benefits may be age-dependent, as demonstrated by the results of a secondary analysis by Basch *et al*.^28,29,42^ This analysis reported a significantly lower risk of emergency room visits and improved survival among younger patients with solid tumours (median age = 58 years, range 26–69 years) whose symptoms were managed electronically in addition to receiving usual care.^42^ These benefits were not observed among older patients who were managed electronically in addition to usual care (median age = 75 years, range 70–91 years).^42^

### Efficient utilisation of healthcare resources

ePROM systems provide medical teams with the opportunity to interact with patients and deliver care efficiently. Changes in patients’ clinical status can be monitored remotely and hospital appointments reserved for those that require in-person assessment. Therefore, the use of ePROMs in the routine care of patients with chronic diseases could lead to a more efficient utilisation of limited healthcare resources. In Denmark, the use of a generic ePROM system, Ambuflex, is being used routinely to manage patients across nine chronic conditions (epilepsy, coronary heart disease, narcolepsy, sleep apnoea, prostate cancer, asthma, rheumatoid arthritis, colorectal cancer and kidney failure) to facilitate clinical decision-making.^24^ Patients complete ePROMs remotely and their data is used to determine whether they require an outpatient hospital appointment. The use of the Ambuflex system led to decreases of 48% and 57% in hospital follow-up visits in patients with epilepsy and sleep apnoea, respectively.^27^

The use of this ePROM system across the chronic conditions also led to reductions in (a) the reimbursement of patient transportation costs and (b) the need to destroy excess chemotherapy drugs for the oncology patients (which might have been due to better treatment adherence).

The previously mentioned study that utilised the STAR ePROM system also reported benefits related to resource utilisation.^28^ After a year of follow up, patients in the STAR arm had significantly fewer emergency department (ED) visits compared with those who received usual care (34% *versus* 41%).^28^ This effect was more pronounced when the computer-inexperienced subgroups of the two study arms were compared directly (34% *versus* 56%). The study also reported a significant reduction in hospitalisation among the computer-inexperienced subgroup of the STAR arm (44% *versus* 63%) but not in the computer-experienced subgroup.^28^

Conversely, in the United Kingdom (UK), the RCT of the eRAPID system by Absolom *et al.* did not find any significant difference in the utilisation of healthcare resources between the intervention and control arms.^33^ Specifically, there were no significant differences in chemotherapy delivery, hospital admissions, acute oncology assessments or emergency hotline calls.^33^

### Improved symptom management

An RCT conducted in Sweden evaluated the utility of an interactive ‘Interaktor’ ePROM app for delivering treatment-related symptom management *versus* usual care for patients with breast cancer receiving chemotherapy.^37^ Patients in the Interaktor group experienced nausea, vomiting, sadness, loss of appetite and constipation less frequently.^37^ Overall physical symptom distress was significantly lower and emotional functioning significantly improved in the Interaktor group (see Table 1).^37^

De Thurah *et al.* conducted an RCT comparing disease activity control for rheumatic arthritis using the AmbuFlex system with usual outpatient follow-up care by physicians. Patients with low disease activity who used AmbuFlex experienced similar levels of disease activity control compared with those who received conventional outpatient follow up.^31^ Furthermore, aside from the fixed yearly outpatient visits, patients randomised to AmbuFlex required fewer extra visits per year (see Table 1).^31^

Based on functional assessment of cancer therapy-general, physical well-being subscale (FACT-PWB) data, there was better symptom control among patients undergoing chemotherapy in the intervention arm of the eRAPID RCT at 6 (*p* = 0.028) and 12 (*p* = 0.039) weeks.^33^ However, no significant difference was recorded at 18 weeks between the two arms of the study (*p* = 0.699).^33^

Mooney *et al.* evaluated the efficacy of an automated symptom management system in patients commencing chemotherapy in an RCT.^41^ All the patients reported their chemotherapy-related symptoms using the system but patients in the symptom care at home (SCH) arm received self-management information and nurse practitioners acted on alerts of poorly controlled symptoms following decision support guidance.^41^ At the end of the follow-up period, symptom severity across all symptoms was significantly less in patients randomised to the SCH arm compared with usual care (*p* < 0.001).

An analysis of PROMs and physician-reported outcomes data, collected as part of an RCT comparing two hypofractionated radiotherapy schedules, was conducted by Rammant *et al*.^46^ The study found poor concordance between patient and physician reported side effects with physicians significantly underrecognising and underreporting patients’ symptom burden.^46^

Handa *et al.* conducted an RCT to evaluate the effectiveness of a smartphone-based application to track treatment side effects of patients undergoing chemotherapy.^38^ In addition to measuring physical symptoms, the application measured changes in patients’ anxiety and depression levels using the hospital anxiety and depression scale (HADS). The study found that medical staff underestimated the severity of 25% of patients’ physical symptoms and the most frequently underestimated were muscle/joint pain, fatigue, and nausea. Symptom monitoring in this study did not lead to significant improvements in patients’ HADS scores.

The studies by Rammant and Handa confirm previous findings that medical teams underestimate symptoms compared with patient reports.^47,48^ They also highlight the need for a mechanism, such as ePROMs, by which patients can provide assessments of their health status to complement clinician assessments.

In an RCT conducted by Kroenke *et al.*, patients who screened positive for at least one SPADE (sleep, pain, anxiety, depression and low energy/fatigue) symptom were recruited (their underlying chronic conditions were unspecified).^40^ They completed the five matching domains of the patient reported outcome measure information system (PROMIS) profile-29 electronically and were subsequently randomised to a feedback group in which their clinician only received a visual display of their symptom scores or a control group in which the scores were not provided to clinicians.^40^ At the end of the 3-month follow-up period, while both groups had moderate symptom improvement, there was a non-significant trend favouring the feedback compared with the control group (see Table 1).^40^

There is a possibility that participants in the RCTs by Handa and Kroenke did not experience a significant improvement in their symptoms because there were no specific interventions to address issues highlighted by the ePROM systems.^38,40,49^ While this might be acceptable for an RCT, depending on the study design and aims, collecting and analysing PROM data in a routine setting without acting on the information by providing treatment/support for problems identified could be a waste of resources.^49^ Kroenke *et al.* concluded that providing clinicians ePROM data without additional systems support or incentives is insufficient.^40^ Medical teams need to carefully consider how ePROMs data will be translated into actionable plans for patient management when developing ePROM systems.

### Improved treatment adherence and monitoring

A multi-centre, non-randomised prospective cohort analysis performed in Belgium compared ePROMs collection using a web-based system (AMTRA) with usual care among patients with solid tumours undergoing chemotherapy.^43^ Patients in the intervention arm used an application to provide daily reports of the severity of their symptoms.^43^ They were allowed to provide additional reports if required. Self-care information was provided for mild or moderate symptoms, and alerts were sent to the medical staff in cases of severe adverse events. Patients experienced a statistically significant reduction of mean severity grade for nausea, constipation, loss of appetite, fatigue and dyspnea over the follow-up period.^43^

The AMTRA app uniquely incorporated a treatment adherence aid that required patients to log their medication intake. An automatic reminder was sent to the provider if an entry was not logged and followed up within 24 h if there was still no response. There was a median treatment adherence rate of 98.7% in the 44 patients receiving oral chemotherapy.^43^ The study’s success may also be attributed to the training that was provided to patients and medical staff prior to study commencement. Medical staff received training on the system and the pathways for responding to patient-generated alerts.^43^ Patients had home visits during which they were shown how to use the application by trainers who emphasised the importance of reporting symptoms and adhering to treatment.

### Reduced risk of disease transmission during disease outbreaks

The COVID-19 pandemic has emphasised the need and relevance of ePROMs in clinical trials and routine practice.^50^ There is now a renewed interest in all forms of telemedicine and the effect will probably remain even after the threat of COVID-19 has subsided.

When the COVID-19 epidemic began, it quickly became apparent that providing medical care outside hospital settings reduced the risk of exposure to, and transmission of, the SARS-CoV-2 virus for patients and clinicians.^50^ Kricke *et al.* recently reported early findings from the implementation of an outpatient COVID-19 monitoring program in the US. Over 6000 patients with presumed COVID-19 infection completed an electronic daily symptom questionnaire from home and were stratified based on symptom severity. While the majority of the patients did not require hospitalisation, remote monitoring led to emergency department referrals for an average of nine patients per day.^39^ With the growing incidence of long COVID, the use of ePROMs for symptom monitoring could facilitate the identification of life-threatening complications that may develop later in patients.

## Challenges with the use of ePROMs

While the studies included in this review demonstrate potential benefits of using ePROMs in patient care, there are a number of challenges that need to be adequately addressed to ensure seamless implementation and integration of ePROMs into a health system.^51^

### Maintaining high ePROM completion rates

The use of ePROMs could precipitate patients’ experiences of survey fatigue, which may lead to a steady decline in survey completion over time.^52^ Other individual and system level factors which may be associated with reduced propensity to complete surveys or to higher survey fatigue, include: older age, disease severity, the presence of comorbidities,^11^ questionnaire length and item relevance, and perceptions of response burden.^53^ Participant fatigue may be minimised with innovative PROM assessment methods. For instance, computerised adaptive testing (CAT), an algorithm-based method, tailors ePROMs to the individual by automatically selecting and sequentially administering the most relevant items (questions) from an item bank based on a respondent’s prior responses.^54–56^ With the expansion of ePROMs, the expansion of CATs and other methods to improve survey design and reduce survey burden should be considered.^57,58^

The AmbuFlex system attributed its high completion rates to the use of a mixed-mode (paper and web) method of data collection.^27^ A recent publication by Niels Hjollund reflected on the 15-year use of the AmbuFlex system for the follow-up of patients with chronic diseases. He noted that although a mixed-mode method of collection of PROM data was initially implemented to maximise response rates (66.5% of responses were paper-based in 2005), there has been a gradual preference for an electronic option (only 4.3% were paper-based in 2019).^59^

Despite a decline from an initial baseline completion rate of 94%, Taarnhoj *et al.* still reported relatively high completion rates of more than 70% over six cycles of chemo- or immunotherapy. The rates only declined after patients had received the sixth cycle of treatment.^60^ In the study by Kricke *et al.*, 20% of patients monitored for COVID-19 symptoms did not fill out the daily questionnaire, particularly those with mild symptoms.^39^ Missing data is also a common problem with patient-reported data. Identifying causes of missing entries could improve the clinical utility of ePROMs and highlight opportunities to improve ePROM completion.^61^

The level of health literacy among patients may significantly influence their decisions to engage with ePROM interventions.^62^ To encourage compliance, efforts need to be made to ensure that patients understand the importance and potential benefits of providing ePROM data in relation to their clinical management and health outcomes.

### Cost and cost-effectiveness

The issues of cost and cost-effectiveness could significantly influence the decision by health care providers to commission the development and implementation of ePROM systems. For policymakers, the cost and cost-effectiveness of ePROM interventions in comparison with existing follow up care may determine whether crucial governmental and/or institutional support in terms of legislation or finance is secured.

Data from a recent trial compared the cost-effectiveness of usual care, tele-rehabilitation, and tele-rehabilitation plus pharmacological pain management for patients with late-stage cancer (details about cancer types not provided).^63^ At the $100,000.00 willingness-to-pay threshold, the tele-rehabilitation model was the most cost-effective strategy in 95% of simulations.^63^ The authors attributed the cost savings to the fact that patients in the tele-rehabilitation arms were less likely to require intensive care unit admission.^63^

Cost-effectiveness analysis was pre-specified as a secondary outcome in an RCT by Denis *et al.*^35^ exploring the impact of ePROMs on survival in patients with lung cancer. The ePROM results for patients in the intervention arm determined the frequency/need for computerised tomography (CT) scans while patients in the control (usual care) arm had CT scans at fixed intervals. The ePROM option was cheaper per patient (941 euros/year/patient) compared with usual care (1304 euros/year/patient). It provided an incremental cost-effectiveness ratio of 12,127 euros per life-year gained and 20,912 euros per quality-adjusted-life-year (QALY) gained. The probabilities that this ePROM option was very cost-effective and cost-effective were 97% and 100%, respectively.^64^

A third study modelled the cost-effectiveness of an ePROM for symptom monitoring in patients undergoing treatment for advanced or metastatic cancer in Alberta, Canada, compared with standard symptom monitoring.^65^ The ePROM system provided 2.17 QALYs at a total cost of $69,030 Canadian dollars (CAD) compared with standard monitoring, which yielded 1.92 QALYs at a total cost of $65, 670 CAD.^65^ A probabilistic sensitivity analysis of 14 variables over 1000 iterations gave a probabilistic mean incremental cost effectiveness ratio (ICER) of $13,110 for the ePROM option and the authors concluded that this option was value for money.^65^

### Scepticism among healthcare professionals

A number of recent studies have explored in-depth the practice tensions, scepticism and divergent views among healthcare professionals (HCPs) regarding the use of PROMs and ePROMs in clinical care.^11,66–68^ Concerns about workload; individual values, beliefs and priorities; lack of specific competence dealing with issues relating to emotional problems; PROM standardisation and quantification were noted as some of the determinants of HCP attitudes.^11,66–68^ A recent meta-synthesis by Easpiag *et al.* showed an encouraging overall positive polarity of opinions among HCPs.^69^

An awareness of the tensions and challenges experienced by HCPs with ePROMs and their engagement and involvement in ePROM system development, implementation and integration is essential to overcome these barriers.^11,66–68,70,71^ Clear guidelines or actionable plans are essential to enable clinicians respond confidently and effectively to ePROM data.^38,40,41,49^

### Integration of ePROMs into existing health systems

The successful integration of ePROM into existing health systems and workflows has the potential to transform clinical practice.^72^ This process may be complex, resource-intensive, requiring an iterative approach and stakeholder involvement.^51,70,72–75^ Appropriate training of HCPs to handle and respond to ePROM data needs to be provided in order to facilitate integration with existing clinical workflows.^43,66,76^ LeRouge *et al.* have recently published a comprehensive toolkit to facilitate the process of implementation and integration of ePROMs with existing health systems.^77^ This toolkit comprises of guidelines that focus on the various aspects of the process including ePROM integration approaches, workflow designing, leveraging of health ICT, and display of data.^77^

## Ongoing ePROM initiatives

There is growing evidence that the thoughtful incorporation of ePROMs into health systems could have meaningful and positive impacts on clinical outcomes. However, most of this evidence to support their use originates from the field of oncology. Thus, there is a need to demonstrate its potential impact in other chronic conditions.

Initial work is underway to explore the use of ePROMs in the routine care of patients with CKD. The RePROM study is currently being conducted by researchers and clinicians based at the Centre for Patient-Reported Outcomes Research at the University of Birmingham and the Queen Elizabeth Hospital Birmingham within the UK National Health Service University Hospitals Birmingham Foundation Trust (UHBFT).^78^ The study will determine the feasibility of undertaking a full-scale RCT of ePROMs in the symptom monitoring of patients living with advanced CKD.^78^

A pilot study also involving patients with CKD at the University Health Network, Toronto, Canada, will test the feasibility of implementing an ePROM toolkit to assess physical and emotional symptoms, using the PROMIS CATs. This is linked to a symptom management, self-management support resource hub.^79,80^

The Evaluation of Routinely Measured Patient-reported Outcomes in Haemodialysis Care (EMPATHY) RCT, which aims to determine the effects of routine measurement of ePROMs on the experiences of patients undergoing in-centre haemodialysis in Alberta and Ontario, Canada, is currently underway.^81^ The use of ePROMs in the management of patients receiving home dialysis is also being trialled in the ePRO Kidney study.^82^

## Conclusions

There is increasing evidence that the use of ePROMs could have significant impacts on outcomes valued by patients, healthcare providers and researchers. While our paper focusses primarily on quantifiable effects of ePROMs on traditional clinical outcomes, this does not mean that other outcomes are less important.^83^

As most of the evidence we found was derived from oncology, there is an urgent need for similar work in other subspecialties that care for medically complex patients. ePROM systems that demonstrate maximum clinical utility and minimum patient burden need to be seamlessly integrated into a learning health system. Whilst the development and implementation of these systems may be initially costly and resource-intensive, patient preferences and existing evidence to support their implementation suggests the need for continued research prioritisation in this area. In addition, there is a need for case studies to demonstrate best practices for achieving and maintaining patient and HCP engagement. Finally, further research is required to demonstrate the long-term cost-effectiveness of using ePROMs in routine clinical practice. Significant work remains, but the implementation of ePROMs provides a valuable opportunity to transform the quality and delivery of care for medically complex patients.
